# Moving Forward in Nano-Immune Interactions

**DOI:** 10.3390/nano12122033

**Published:** 2022-06-13

**Authors:** Giuseppe Bardi, Monica Neagu

**Affiliations:** 1 Nanobiointeractions & Nanodiagnostics, Istituto Italiano di Tecnologia, Via Morego 30, 16163 Genova, Italy; 2 “Victor Babes”, National Institute of Pathology, 99-101 Spl Independentei, 050096 Bucharest, Romania

This is the second Special Issue on the topic “Immune Responses to Nanomaterials for Biomedical Applications”. The first hosted eleven papers, including research articles and reviews, achieving a high citation score [1,2].

Nanomaterials have various effects in the immune system. Researchers have tailored their nano-tools to stimulate or decrease innate and adaptive responses in the fight against microorganisms and the uncontrolled growth of tumor cells.

We decided to publish a follow-up on this field as new nanotechnologies for immune applications are continuously appearing. Some of them have already been exploited in vaccine formulations to counteract the effects of COVID-19 [3], while others are still on the bench or are potentially applicable [4]. The successful application of nanoparticles as vaccine carriers is undoubted. Nevertheless, concerns regarding the chemical surface of nanoparticles with immune cells or inflammatory mediators have been raised. Polyethylene glycol (PEG) moieties are especially helpful in delaying blood clearance, but have recently attracted medical attention for their cross-reactivity and immediate reactions [5]. Nano-immune interactions are therefore the core topic of this Special Issue. Antibody-complementary complexes and the interference of nanomaterials with normal immune responses is another area of active investigation that will be gladly peer-reviewed here.

Immunology will incessantly benefit from the new synthesis and characterization of several nanomaterials that are difficult to describe in one single use. Although seminal applications of nanoparticles have focused on the role of nanomaterials as drug delivery carriers, today, novel synthesized nanomaterials offer countless opportunities as genetic material carriers, intrinsic therapeutic tools or diagnostic sensors. We believe that the wide-ranging potential of nanotechnologies related to immunology will be crucial in the future, whether for delivery with the use of inorganic or organic nanoparticles, for specific therapeutic release using nanogels or polymer nanocages, or for signal amplification in order detect picomolar concentrations of inflammatory markers using metallic nanozimes.

As with the previous issue [1], the mechanisms of immune responses to nanomaterials and their toxicological profile remain the main pillars of the topic. Cytotoxicity is the endpoint of many immune responses. Defining the adverse outcome pathways (AOP) of the different nanomaterials released in specific tissues is a fundamental argument welcomed in the present Special Issue. Our group is deeply involved in the immune-toxicological characterization of nanoparticles [6,7], and we are convinced that comprehensive reviews are essential to discuss the results emerging from different observations and to define new scenarios from a wider perspective. Furthermore, review articles are a major source of guided information for students and young researchers to gain knowledge and plan innovative experiments.

We also encourage updates of previous studies from research groups with clear expertise in a specific topic. Improvements in selective nanoparticle targeting [8], metal or peptide nanoparticle inflammatory response [9,10] and combined therapies [11] have already been published in the previous issue, but the present one could certainly benefit from further results and updates.

Methodologies and technical developments using nanomaterials [6,12] are very important in sharing knowledge among scientists and we encourage submissions on this subject in particular. Indeed, improvements in sensing inflammatory mediators, including cytokines and chemokines, is a hot topic in the field [13]. Portable assays are gaining more and more public and marketing consideration. Lateral Flow Assays (LFA) for point-of-care immune detection often require “smart” nanomaterials to amplify the recognition signals and give a clear response. This area of research is fast-growing, and we are keen to revise papers on this subject.

In the hope of contributing positively to the knowledge of nanomaterial-immune system interactions, we hope our readers find the articles presented in this Special Issue to be helpful and informative.
